# Effects of *SpsNAC042* transgenic *Populus hopeiensis* on root development, leaf morphology and stress resistance

**DOI:** 10.1270/jsbbs.22079

**Published:** 2023-04-13

**Authors:** Lijiao Fan, Dongshan Wei, Xingwang Yu, Fengqiang Yu, Jiameng Wang, Guirong Sun, Alatengsuhe, Li Zhang, Guosheng Zhang, Haifeng Yang

**Affiliations:** 1 College of Forestry, Inner Mongolia Agricultural University, Hohhot 010018, China; 2 Development Center of Forestry and Grassland, Ordos 017000, China; 3 General Headquarters of Ordos Afforestation, Ordos 017000, China

**Keywords:** *SpsNAC042* gene, stress, tolerance

## Abstract

To identify the function of the *SpsNAC042* gene and its response to salt and drought stress, the *SpsNAC042* gene was transformed into *Populus hopeiensis* by the Agrobacterium-mediated leaf disc method, and the phenotypic, physiological changes and related genes expression of transgenic lines were analyzed. The results showed that the number and length of roots of transgenic lines increased significantly. The leaves of transgenic lines curled inward. Under salt and simulated drought stress, the transgenic lines showed improved tolerance to salt and drought. The activities of SOD, POD, CAT and proline content in the transgenic lines were significantly increased, and the reduction rates of total chlorophyll and MDA content were significantly decreased, which indicated that the transgenic lines showed strong physiological responses under stress. Meanwhile, the gene expression of *MPK6*, *SOS1*, *HKT1* and *P5CS1* were significantly upregulated, and the gene expression of *PRODH1* was significantly downregulated, which preliminarily verified the stress regulation mechanism that *SpsNAC042* might activate. The above results showed that the *SpsNAC042* gene could promote root development, make leaf morphology curl, and enhance *P. hopeiensis* tolerance to stress.

## Introduction

*NAC* is one of the unique transcription factor families unique to higher plants. The name of *NAC* family is composed of the first letters of *NAM* (no apical meristem) (Souer *et al.* 1996), *ATAF1/2* and *CUC* (cup-shaped cotyledon) (Aida *et al.* 1997). Among this gene family, *NAM* was cloned from *Petunia hybrida* primitively, and *ATAF1/2* and *CUC2* were found in *Arabidopsis*. In recent years, more *NAC* transcription factors have been identified in *Oryza sativa* (Ooka *et al.* 2003), *Arabidopsis* (Nuruzzaman *et al.* 2010), *Nicotiana tabacum* (Rushton *et al.* 2008), *Glycine max* (Le *et al.* 2011) and other plants. Through statistical analysis, they were subdivided into different subfamilies, providing a research basis for future exploration and validation of the *NAC* family. Studies have shown that *NAC* transcription factors play an important regulatory role in plant growth and development (Olsen *et al.* 2005), and are also involved in abiotic stress responses of plants to drought, salinity and low temperature (Sun *et al.* 2012). Whole-genome identification of *NAC* transcription factors was performed in many plants, including cultivated peanuts and two wild ancestral species of peanut, *Arachis duranensis* and *Arachis ipaensis*. At the same time, the stress response-related *NAC* transcription factors *AhNAC127*, *AhNAC87* and *AhNAC117* were screened from RNA-seq data in cultivated peanuts, and were identified in response to drought and salt stress (Lu 2020). The *NAC1* gene of *Arabidopsis* is a homologous sequence of *SpsNAC042*, and the function of *NAC1* gene in more species has been gradually verified (Yu 2020). The stress-responsive *NAC* transcription factor gene *GhSNAC1* cloned from *Gossypium hirsutum* was transformed into *Nicotiana tabacum*, and the drought and salt tolerance of transgenic lines were significantly improved (Wang *et al.* 2019). *MdNAC1* was cloned in *Malus domestica* *‘Golden Delicious’*, and overexpression of this gene in GL-3 apple enhanced the antioxidant capacity of the plant under drought stress conditions and alleviated the damage caused by drought stress suffered by the plant. The *MdNAC1* expression in *Lycopersicon esculentum* similarly enhanced the resistance of the transgenic lines to drought stress (Jia 2019). *Arabidopsis* with *VvNAC1* overexpressing exhibit enhanced tolerance to osmotic, salt and cold stresses (Le Hénanff *et al.* 2013). At the transcriptional level, the expression of *TaNAC1* gene was induced by abiotic stress conditions such as PEG, ABA, low temperature and high salt (Ai *et al.* 2016). By heterologously expressing of the pumpkin transcription factor *CmoNAC1* in *Arabidopsis*, it was found that transgenic lines got better salt tolerance (Cao 2020). With the more research works on abiotic stress resistance, the more stress resistance genes were verified in many plants.

Research on transgenic salt-tolerant and drought-tolerant trees is an effective way to deal with soil salinization and desertification. The stress resistance research on woody plants has become an important reference for afforestation in frigid regions as to ecological construction. In 1994, salt-tolerant transgenic poplar was first obtained in China (Tian and Tan 2009). In 2000, the salt-tolerant gene *mtl-D* was transferred into *Poplar* × *xiaozhannica* and obtained the first batch of transgenic plants with high salt tolerance (Liu *et al.* 2000). In 2006, through the field release afforestation test, it was proven that the growth of *Poplar* × *xiaozhannica* was significantly increased and showed tough traits in medium saline soil, and it could be widely used in saline soil environmental governance (Yin *et al.* 2006). In 2014, the *ZxZF* transcription factor gene acted a significant role in improving the drought resistance of transgenic poplar and had important breeding value in cultivating excellent varieties of drought-resistant trees (Zhang *et al.* 2014). The overexpression of the *chlAPX* gene in *Populus tomentosa* showed higher stress resistance (antioxidant, drought resistance, salt tolerance) (Lang 2014). In 2015, *P. simonii* × *P. euphratica ‘Liaohu 1’* and *P. simonii* × *P. euphratica ‘Liaohu 2’* obtained by hybridization were tolerant to medium and mild saline alkali (Wang and Peng 2015a, 2015b). At present, the distribution of arid or semiarid regions in China has seriously hindered the development of the ecological environment, and research on drought-resistant and salt-resistant plants is of great importance.

*Populus hopeiensis* is a unique natural hybrid of *Populus davidiana* × *P. tomentosa* in China. It is one of the main afforestation tree species in gully region and sandy region of the Loess Plateau, and is also an important tree species for soil and water conservation and timber forests (Liu 1998). However, the application of *P. hopeiensis* in some fragile habitats in northern China was still restricted. The tolerance of *P. hopeiensis* to adversity should be further improved, which is conducive to its great role in ecological construction and shelter forest construction. *Salix psammophila* is an important shrub species for sand-fixation afforestation in Northwest China and own the characteristics of drought resistance, salt-alkali resistance and sand resistance (Lu *et al.* 2020). In our study, the *NAC042* gene isolated from *S. psammophila* was transformed into *P. hopeiensis*, and the phenotypic physiological and biochemical changes of transgenic lines under salt and simulated drought stress were analyzed to confirm their stress resistance.

## Materials and Methods

### Evolutionary tree construction of genes in *Salix*, *Arabidopsis*, and *Populus*

The complete genome annotation information and sequences of *Salix*, *Arabidopsis*, and *Populus* were obtained from the Phytozome database (https://phytozome-next.jgi.doe.gov/). Multiple sequence alignment of NAC proteins was carried out using the Clustal Omega online (https://www.ebi.ac.uk/Tools/msa/clustalo/). The phylogenetic tree was constructed with MEGA X (MEGA X 10.2.5) using the neighbor-joining (NJ) method with 1000 bootstrap test replicates.

### Experimental materials and vector construction

*S. psammophila* was sampled at the Germplasm bank of *S. psammophila* in Ordos Dalad, the Inner Mongolia Autonomous Region of China. The 1-year-old branches of *S. psammophila* clone (No. 11-30) were collected to clone the *SpsNAC042* gene. The tissue culture seedlings of *P. hopeiensis* were cultured in the forest genetics and breeding department of Forestry College, Inner Mongolia Agricultural University. The *SpsNAC042* gene was cloned into the overexpression vector pMDC32 driven by the 2 × 35s promoter and transformed into *P. hopeiensis* (Yu 2020). Agrobacterium tumefaciens strain GV3101 carrying the CaMV 35S::*SpsNAC042* plasmid was stored in a refrigerator at –80°C.

### Agrobacterium-mediated transformation of *P. hopeiensis* leaf discs and PCR assay and relative expression levels of *SpsNAC042* in positive lines

Agrobacterium tumefaciens containing the 35S:*SpsNAC042* recombinant plasmid was activated and transformed into wild-type *P. hopeiensis* by leaf discs (Horsch *et al.* 1985, Yu 2020). The CTAB method (Chen *et al.* 2004) was used to extract total DNA from leaves of resistant *P. hopeiensis*. Based on the CDS of *SpsNAC042*, the optimal primers were designed on primer 5 as *SpsNAC042-F* and *SpsNAC042-R* (Table 1). The *SpsNAC042* gene fragment was amplified by PCR. The PCR amplification procedure was 94°C for 5 min, 35 cycles (94°C for 30 s, 62°C for 30 s, and 72°C for 1 min), and 72°C for 7 min. The samples were tested with 1% agarose gel.

The whole plants of 17 positive lines (including shoots, leaves, and stems) were collected. RNA was extracted from plant samples by RNAprep Pure Plant Plus Kit (Polysaccharides & Polyphenolics-rich) (Tiangen, China). Complementary DNA (cDNA) is synthesized from the RNA template with a Super Script IV First-Strand Synthesis System kit (Invitrogen, USA). Real-time fluorescence quantitative PCR (RT-qPCR) was performed on a Roche Light Cycler 480 II with SYBR^®^ Premix Ex TaqTM II (Tli RNaseH Plus). Each reaction was repeated three times and the quantitative data were calculated by 2^–ΔΔCt^ method (Livak and Schmittgen 2001).

### Tissue-specific expression of *SpsNAC042* in *S. psammophila* and relative expression levels of *SOS1*, *MPK6*, *HKT1*, *P5CS1* and *PRODH1* in overexpression lines

Six different tissue parts of *S. psammophila*, including leaves, roots, shoots, soft stems, semi-lignified stems and mature stems, were collected. RNA was extracted from the six different tissue parts of *S. psammophila* and reverse transcribed to cDNA, and qRT-PCR was performed to detect expression of the *SpsNAC042* gene.

The primers for quantitative detection of the *SpsNAC042* gene were *qN1-F* and *qN1-R*, and the primers for the internal reference gene were *UBQ-F* and *UBQ-R* (Yang *et al.* 2021). The primers for the internal reference gene of *P. hopeiensis* were *ACT-F* and *ACT-R* (Table 1).

The expressions of the *SOS1*, *MPK6*, *HKT1*, *P5CS1* and *PRODH1* genes under different treatments were detected via qRT-PCR. The primers for gene quantification were *SOS1-F1*, *SOS1-R1*, *MPK6-F1*, *MPK6-R1*, *HKT1-F1*, *HKT1-R1*, *P5CS1-F1*, *P5CS1-R1*, *PRODH1-F1* and *PRODH1-R1* (Table 1), and the internal reference gene primer for the *P. hopeiensis* genes were *ACT-F* and *ACT-R*. The primer sequences of the *SOS1*, *MPK6*, *HKT1*, *P5CS1* and *PRODH1* genes were designed by Primer 3 input (Whitehead Institute for Biomedical Research, Massachusetts, USA, https://bioinfo.ut.ee/primer3-0.4.0/).

### Transplantation before plant stress, phenotypic observation and determination of growth indexes

Tissue culture seedlings of transgenic *P. hopeiensis* and wild-type were transferred to the pots, and cultured in the mixed substrate with peat soil and vermiculite (2:1) in it at 25°C with 16 h/8 h light/dark cycle. The seedlings were moved into the greenhouse (25°C) after 2 weeks. The root and leaf morphology of wild-type and transgenic lines cultured for 60 d were observed and counted, and the number of roots, root length and fresh weight were measured. The stress treatment was kept for 7 days, with water as the control group, 0.9% NaCl solution and 10% PEG solution as the experimental group, and each treatment was repeated three times. After 7 days, the phenotypic changes of each line were observed, and the functional leaves (3–6 pieces) of each line were sampled and preserved. Each treatment was repeated three times.

### Salt and drought treatments and determination of total chlorophyll, SOD, POD, CAT, MDA and Pro

The 3 different treatments on the transgenic lines and wild-type, including water, salt and simulated drought stress, were performed and kept for 7 days. The fresh leaves of wild-type and transgenic *P. hopeiensis* lines under water, salt and simulated drought stress were collected and cut into filaments with a width of 2 mm. The filaments were extracted with a mixture of acetone and ethanol (volume ratio 1:1) for 24 h. The absorbance was measured at 663 nm, 645 nm, and 470 nm by spectrophotometer, and the total chlorophyll content was calculated (Zhang 2016). A superoxide dismutase (SOD) activity, peroxidase (POD) activity, catalase (CAT) activity, malondialdehyde (MDA) content, and proline (PRO) content detection kit (BC0170, BC0090, BC0200, BC0020, and BC0290 Solarbio, China).

### Statistical analysis

All statistical data were subjected to three biological replicates. Excel 2019, F-test (two-sample ANOVA), t-test (two-sample equal/heteroscedasticity hypothesis) and one-way ANOVA for randomized block design (Ning 2012). The results of one-way ANOVA for randomized block design are labeled using lowercase letters. Other significance was designated as follows: *** p < 0.001, extremely significant; ** 0.001 ≤ p < 0.01, very significant; * 0.01 ≤ p < 0.05, significant.

## Results

### Functional prediction of the *SpsNAC042* gene

**Fig. 1.**
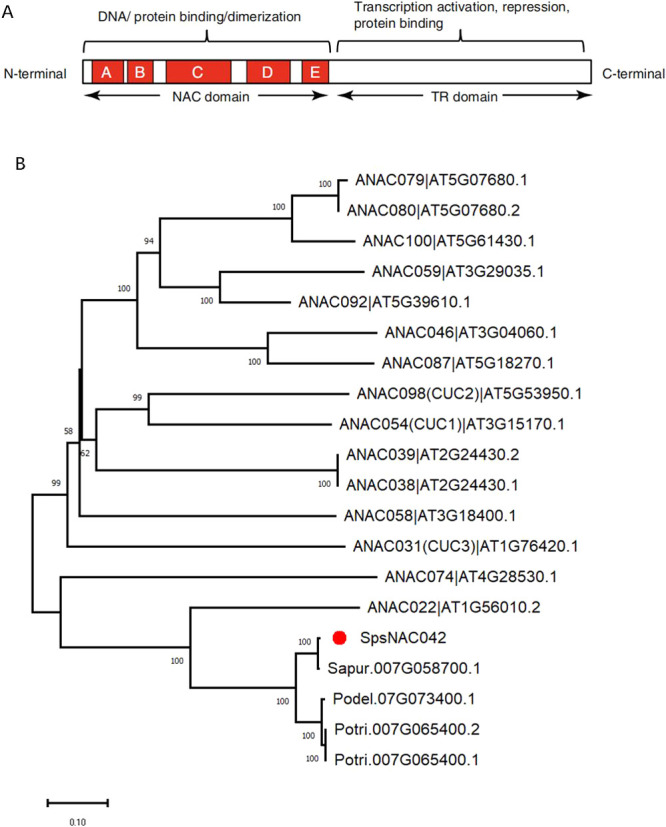
Evolution and protein structure of *SpsNAC042* homologous gene. (A) Schematic diagram of *NAC* transcription factor structure (Puranik *et al.* 2012); (B) Phylogenetic analysis of NAC-a proteins from *Salix*, *Arabidopsis*, and *Populus*.

To preliminarily predict the function of *SpsNAC042* gene, the SpsNAC042 protein motif and comparative analysis of its homologous genes were performed. *SpsNAC042* was cloned from *S. psammophila* and named after *NAC* gene classification in *Populus trichocarpa* (Hu *et al.* 2010). Subdomain A, subdomain B, subdomain C, subdomain D and subdomain E were the typical structural domains of NAC proteins with highly conserved NAM structural domains (Fig. 1A). *SpsNAC042* contains a complete NAM domain (A–E) (Supplemental Fig. 1).

According to the phylogenetic tree of NAC proteins between *Arabidopsis* and *S. psammophila* published by our research group, we found that *SpsNAC042* was clustered into the NAC-a subfamily, and the typical gene in this subfamily was *CUC* (Yang *et al.* 2022). Our study reanalyzed the evolution of some NAC proteins in *Arabidopsis*, *Salix*, and *Populus* based on the clustering of NAC-a subfamilies (Fig. 1B). Transcription factor *CUC* involves in the coordination of ovule formation (Guo *et al.* 2005), which can limit leaf growth through cell cycle inhibition (Li *et al.* 2020), construct plant structure and morphogenesis (Bouré *et al.* 2022), and negatively regulate cell growth factors (Serra and Perrot-Rechenmann 2020). *SpsNAC042* was obtained from homologous cloning of AT1G56010.2 (*NAC1*), Potri.007G065400.1 and Sapur.007G058700.1 (Yu 2020). We can predict the function of *SpsNAC042* gene by homologous genes (Supplemental Fig. 2). Overexpression of *ZmNAC1* in *Arabidopsis* increased lateral roots (Li *et al.* 2012). *Arabidopsis* with *VvNAC1* overexpressing exhibit enhanced tolerance to osmotic, salt and cold stresses (Le Hénanff *et al.* 2013). According to the existing studies on the function of homologous genes, we predicted that *SpsNAC042* gene might affect the growth of plant lateral roots and respond to abiotic stress and stress from some pathogens.

### Tissue-specific expression of the *SpsNAC042* gene in *S. psammophila*

**Fig. 2.**
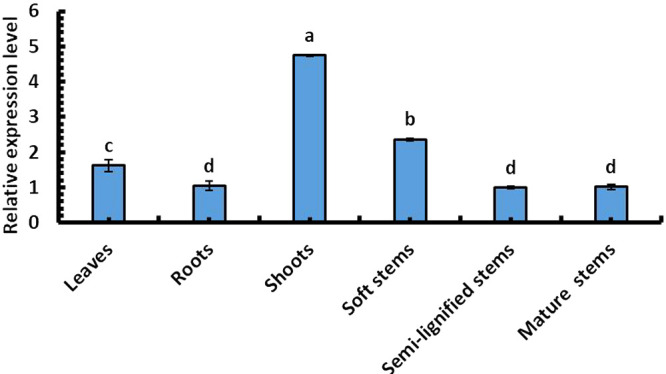
Tissues relative expression level of *SpsNAC042* in *S. psammophila*. Error bars represent ± SD from three biological repeats. These samples used the One-way ANOVA for a randomized block design. As long as there was one same marked letter, the difference was not significant, and the difference was significant if there was a different marked letter. Generally, lowercase letters indicate the significance level α = 0.05.

In order to explore the tissue-specific expression of *SpsNAC042* gene, the relative expression levels of *NAC042* gene in leaves, roots, shoots, soft stems, semi-lignified stems and mature stems were determined. The results showed that *SpsNAC042* had the highest expression level in shoots and the lowest expression level in semi-lignified stems (Fig. 2). Data analysis showed that the relative expression levels of *SpsNAC042* in shoots, leaves and soft stems were significantly different (p < 0.05). However, there was no significant difference among the roots, semi-lignified stems and mature stems among the three tissues (p > 0.05).

### Acquisition of *SpsNAC042* gene overexpression lines, PCR identification, and *SpsNAC042* gene relative expression level in transgenic *P. hopeiensis*

**Fig. 3.**
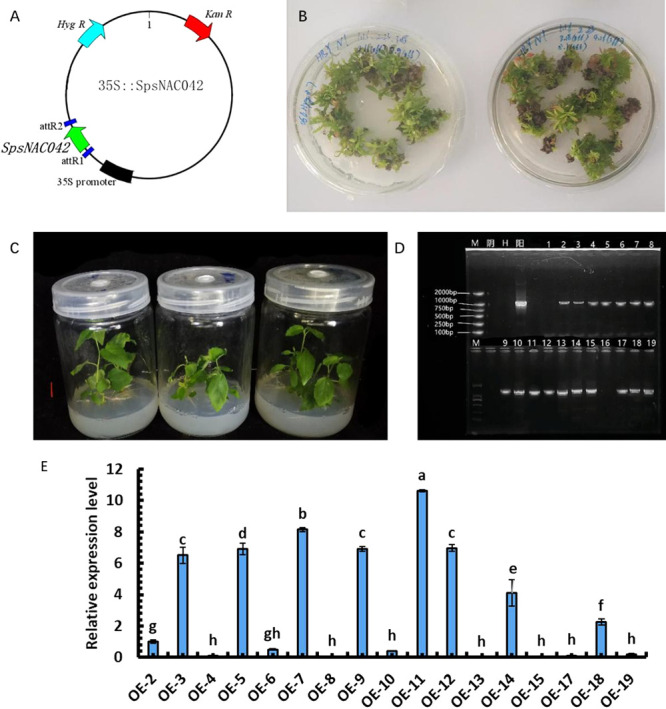
Identification of resistant lines of *P. hopeiensis*. (A) The overexpression vector CaMV 35S::*SpsNAC042*; (B) The resistant shoots of *P. hopeiensis*; (C) The rooting plants of *P. hopeiensis*; (D) Agarose gel assay of PCR amplified target genes from *P. hopeiensis* resistant lines; (E) Determination of relative gene expression in *SpsNAC042* positive lines. These samples used the One-way ANOVA for a randomized block design. As long as there was one same marked letter, the difference was not significant, and the difference was significant if there was a different marked letter. Generally, lowercase letters indicate the significance level α = 0.05.

To obtain *SpsNAC042* gene in overexpression lines, the constructed overexpression vector CaMV 35S::*SpsNAC042* (Fig. 3A) was transformed into wild-type *P. hopeiensis* by the Agrobacterium-mediated leaf disc method. The resistant shoots (Fig. 3B) were obtained by antibiotic screening, and the resistant lines (Fig. 3C) were identified by PCR. The size of the bands obtained by PCR was the same as the expected band size (Fig. 3D).

To select the high-expression transgenic lines, the relative expression level of the *SpsNAC042* gene was analyzed in 17 positive lines of *P. hopeiensis* by qRT-PCR (Fig. 3E). The results showed that the expression of OE-11 was the highest, followed by OE-7 and OE-12. The relative expression levels of OE-3, OE-5, OE-7, OE-9, OE-11, OE-12, OE-15 and OE-18 were significantly higher. The OE-11, OE-7 and OE-12 lines with high expression were selected for stress treatment.

### Changes of root growth in *SpsNAC042* overexpression *P. hopeiensis*

**Fig. 4.**
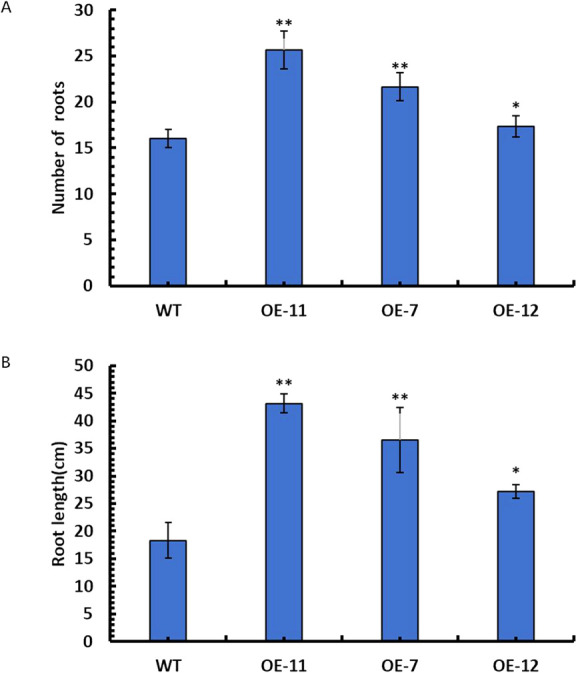
Root growth changes of transgenic *P. hopeiensis*. (A) Statistics of root number of transgenic *P. hopeiensis*; (B) Statistics of root length of transgenic *P. hopeiensis*. (Means ± S.D.) These averages are the average of three individual measurements. The significance of differences was determined based on t-test and F-test. *** p < 0.001, extremely significant; ** 0.001 ≤ p < 0.01, very significant; * 0.01 ≤ p < 0.05, significant.

**Fig. 5.**
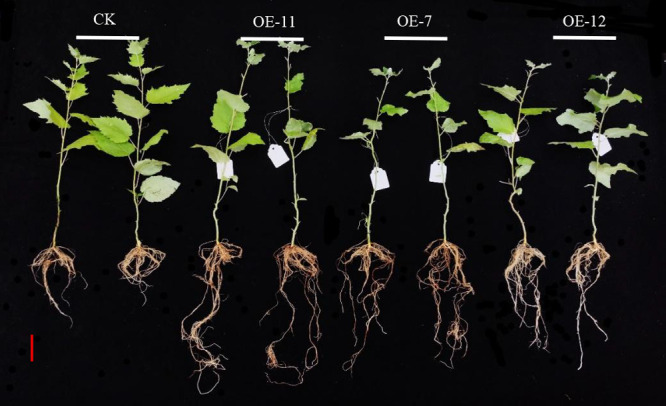
Root status of transgenic *P. hopeiensis*. Root scale = 10 cm.

In order to identify the *SpsNAC042* gene influences on root phenotypic changes, the root’s number and length of OE-11, OE-7, OE-12 and wild-type plants cultured for 60 days were measured. The statistical results showed that the average root number of OE-11, OE-7 and OE-12 increased by 60.42%, 35.42% and 8.33% compared with wild-type (Fig. 4A). The average root length of OE-11, OE-7 and OE-12 increased by 135.45%, 99.09% and 48.18% compared with wild-type (Fig. 4B). The above results showed that the root’s number and length of transgenic lines was significantly higher than those of wild-type (Fig. 5). Among them, the number and length of roots of OE-11 and OE-7 were very significant, and OE-12 was significant.

### Changes of leaf morphology in *SpsNAC042* overexpression *P. hopeiensis*

**Fig. 6.**
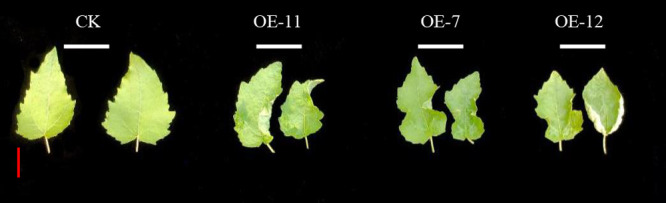
Leaf morphological changes of transgenic *P. hopeiensis*. Scale = 3 cm.

To explore the effects of *SpsNAC042* on leaf morphology, we compared the leaf morphology between transgenic lines and wild-type. The results showed that the mature leaves of OE-11, OE-7 and OE-12 were curled inward and the leaf margin was shrunk, while the leaves of wild-type grown in the same culture environment were flat without curl phenotype (Fig. 6). The results showed that *SpsNAC042* overexpression affected the development of leave morphology.

### Morphological changes of *SpsNAC042*-overexpressing *P. hopeiensis* under stress treatment

**Fig. 7.**
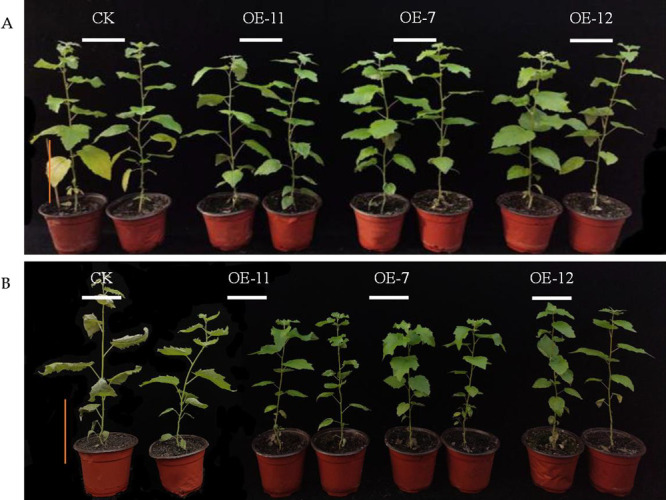
Morphological changes in *P. hopeiensis* under stress. (A) Morphological changes under salt stress; (B) Morphological changes under simulated drought stress, Scale = 10 cm.

To study the effect of stress on plant growth, the transgenic lines and wild-type were subjected to salt and simulated drought stress, and the changes in plant phenotype were observed. 7 days later, the leaves of transgenic lines remained alive under salt stress, but the leaves of wild-type gradually turned yellow from leaf margin to petiole (Fig. 7A). Most leaves of transgenic lines remained alive under simulated drought stress, while the leaves of wild-type turned dehydrated and shrunk (Fig. 7B). The above results showed that the transgenic lines enhanced their tolerance to salt and drought environments.

### The aboveground fresh weight and underground fresh weight of *SpsNAC042*-overexpressing *P. hopeiensis* under stress

**Fig. 8.**
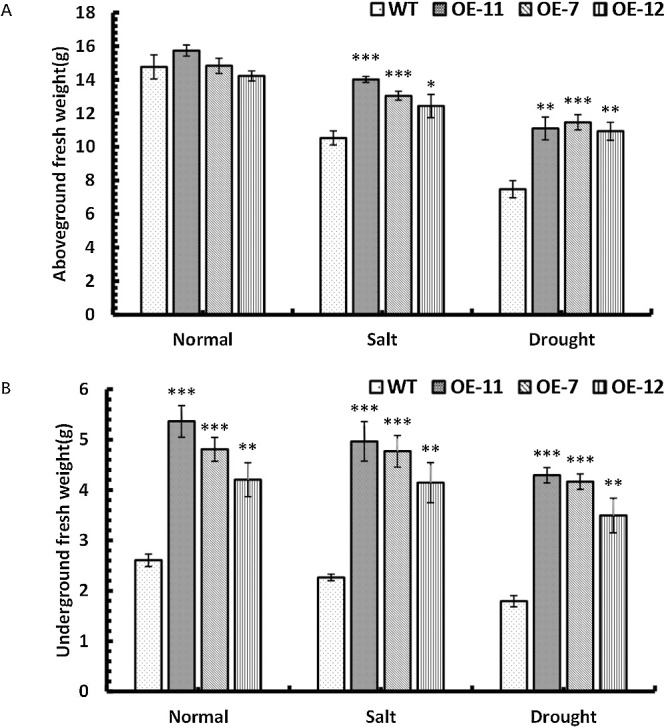
Biomass statistics of transgenic *P. hopeiensis* under stress. (A) The aboveground fresh weight of *P. hopeiensis* under stress; (B) The underground fresh weight of *P. hopeiensis* under stress. (Means ± S.D.) These averages are the average of three individual measurements. The significance of differences was determined based on t-test and F-test. *** p < 0.001, extremely significant; ** 0.001 ≤ p < 0.01, very significant; * 0.01 ≤ p < 0.05, significant.

In order to explore the effect of *SpsNAC042* gene on plant water loss under stress, the aboveground and underground fresh weights of *P. hopeiensis* under stress treatment were measured. The statistical data showed that the aboveground fresh weight of the OE-11, OE-7 and OE-12 was 1.33, 1.24 and 1.18 times higher than that of wild-type under salt treatment. Under simulated drought stress, the aboveground fresh weight of the OE-11, OE-7 and OE-12 was 1.48, 1.53 and 1.46 times higher than that of wild-type. Among them, the aboveground fresh weight of each transgenic line was significantly higher than control group (Fig. 8A). The results showed that the water loss of transgenic lines was lower than that of wild-type under stress, *SpsNAC042* gene reduced the water loss of transgenic lines. The data showed that under salt stress, the underground fresh weight of OE-11, OE-7 and OE-12 was 2.19, 2.11 and 1.83 times than that of wild-type. The underground fresh weight of OE-11, OE-7 and OE-12 was 2.39, 2.32 and 1.95 times than that of wild-type under simulated drought stress. The results showed that the underground biomass of transgenic lines was significantly higher than that of wild-type under stress (Fig. 8B), and this conclusion is consistent with the phenotype of lateral roots significantly increasing in transgenic lines. It was verified again that *SpsNAC042* gene has growth-promoting effect on lateral root development of *P. hopeiensis*, and has positive effect on the increase of underground biomass.

### The total chlorophyll content of *SpsNAC042*-overexpressing *P. hopeiensis* under stress

**Fig. 9.**
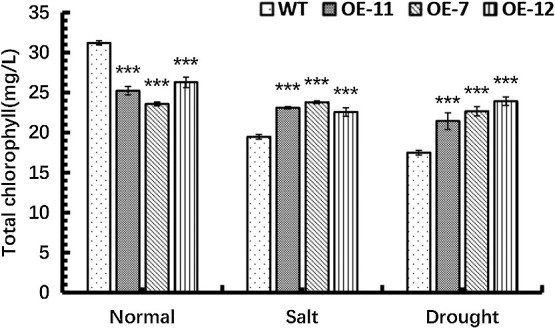
Total chlorophyll content of *P. hopeiensis* under stress. (Means ± S.D.s) These averages are the average of three individual measurements. The significance of differences was determined based on t-test and F-test. *** p < 0.001, extremely significant.

To explore the effect of *SpsNAC042* gene on photosynthesis in transgenic lines under stress, the total chlorophyll contents of *P. hopeiensis* under stress treatment were determined. Non-stress treatment, the total chlorophyll contents were significantly decreased by 19.15%, 24.43% and 15.76% in OE-11, OE-7 and OE-12 compared with wild-type, respectively. Under salt stress, the total chlorophyll contents were significantly increased by 18.68%, 22.25% and 15.93% in OE-11, OE-7 and OE-12 compared with wild-type, respectively. Under simulated drought stress, the total chlorophyll contents were significantly increased by 22.59%, 29.60% and 36.73% in OE-11, OE-7 and OE-12 compared with wild-type, respectively. The results showed that the total chlorophyll contents of transgenic lines increased significantly compared with wild-type under stress, however and the chlorophyll contents of transgenic lines decreased significantly in non-stress treatment (Fig. 9). The results showed that salt and drought stress caused obvious damage to the normal synthesis of chlorophyll in plants, and *SpsNAC042* gene may play protective role in maintaining the normal operation of chlorophyll synthesis or chlorophyll structure in plants to some extent.

### The activities of SOD, POD and CAT of *SpsNAC042*-overexpressing *P. hopeiensis* under stress

**Fig. 10.**
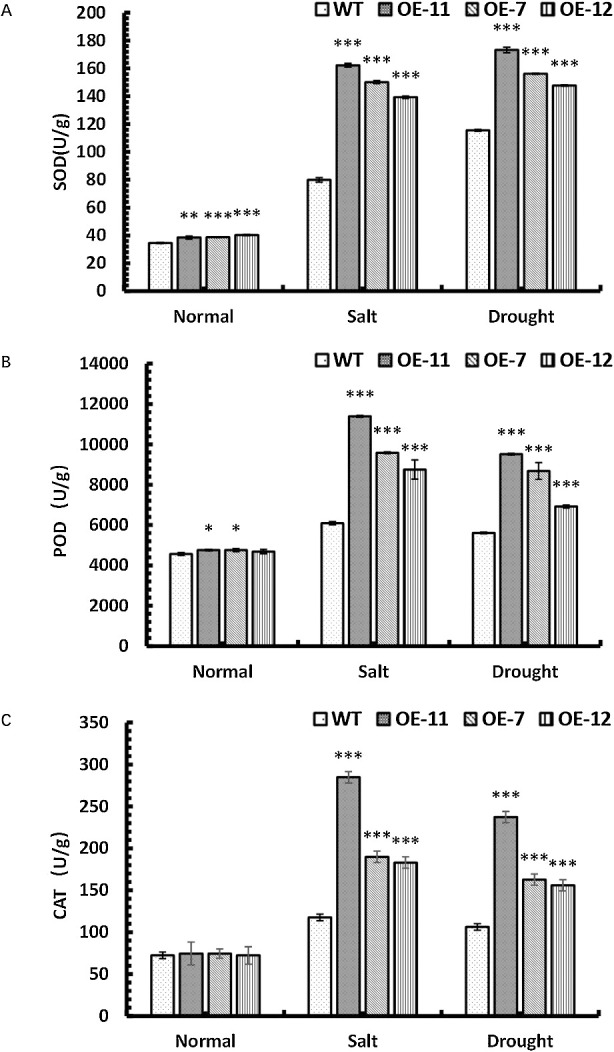
Changes in physiological indicators of *P. hopeiensis* transgenic lines under stress. (A) SOD activities of *P. hopeiensis* under stress; (B) POD activities of *P. hopeiensis* under stress; (C) CAT contents in *P. hopeiensis* under stress treatment. Values are the mean ± standard deviation of three independent experiments. The significance of differences was determined using t-test and F-test. *** p < 0.001, extremely significant; ** 0.001 ≤ p < 0.01, very significant; * 0.01 ≤ p < 0.05, significant.

To confirm the adaptation of transgenic lines to stresses, the SOD, POD and CAT activities of stress-treated plants were determined. Under salt stress, the SOD activities were significantly increased by 103.04%, 87.88% and 74.29% in OE-11, OE-7 and OE-12 compared with wild-type, respectively; the POD activities were significantly increased by 87.11%, 57.42% and 43.75% in OE-11, OE-7 and OE-12 compared with wild-type, respectively; the CAT activities were significantly increased by 142.31%, 61.54% and 55.77% in OE-11, OE-7 and OE-12 compared with wild-type, respectively. Under simulated drought stress, the SOD activities were significantly increased by 50.06%, 35.26% and 27.92% in OE-11, OE-7 and OE-12 compared with wild-type, respectively; the POD activities were significantly increased by 69.49%, 54.66% and 23.31% in OE-11, OE-7 and OE-12 compared with wild-type, respectively; the CAT activities were significantly increased by 123.40%, 53.19% and 46.81% in OE-11, OE-7 and OE-12 compared with wild-type, respectively. The above results showed that the SOD, POD and CAT activities of transgenic lines were significantly higher than those of wild-type under stress (Fig. 10). Meanwhile, *SpsNAC042* can enhance the activity of SOD, POD and CAT to maintain the normal physiological process of transgenic lines under stress and reduce damage to plants.

### Changes of the Pro and MDA contents of *SpsNAC042*-overexpressing *P. hopeiensis* under stress

**Fig. 11.**
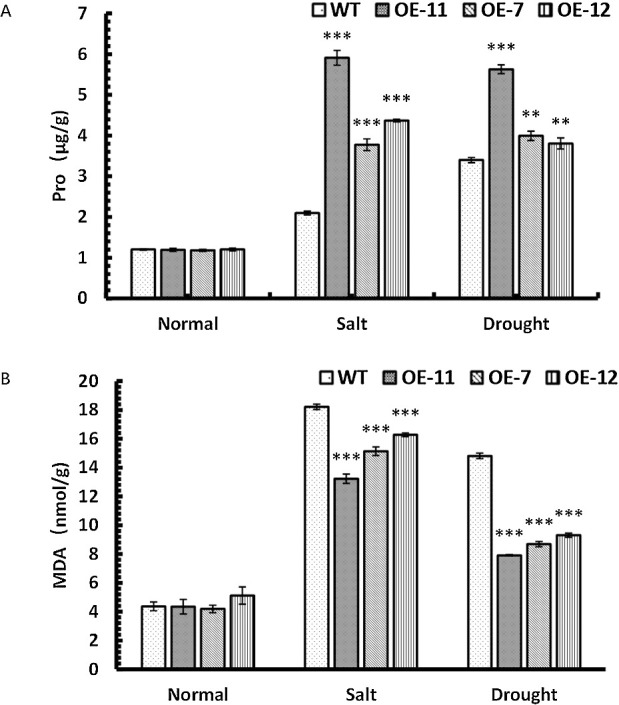
Changes in physiological indicators of *P. hopeiensis* transgenic lines under stress. (A) Pro contents of *P. hopeiensis* under stress; (B) MDA contents in *P. hopeiensis* under stress treatment. Values are the mean ± standard deviation of three independent experiments. The significance of differences was determined using t-test and F-test. *** p < 0.001, extremely significant; ** 0.001 ≤ p < 0.01, very significant; * 0.01 ≤ p < 0.05, significant.

To further explore the damage degree’s differences between transgenic lines and wild-type of *P. hopeiensis* under stress, we measured the content of Pro and MDA in plants. Proline is one of the most important osmoregulatory substances in plants and can scavenge free radicals and improve antioxidant enzyme protection. MDA is the final decomposition product of membrane lipid peroxidation, and its content can reflect the degree of stress injury to plants. Under salt stress, the Pro content were significantly increased by 2.81, 1.80 and 2.08 times in OE-11, OE-7 and OE-12 compared with wild-type, respectively; the MDA content were significantly decreased by 27.39%, 16.88% and 10.63% in OE-11, OE-7 and OE-12 compared with wild-type, respectively. Under simulated drought stress, the Pro content were significantly increased by 1.66, 1.17 and 1.12 times in OE-11, OE-7 and OE-12 compared with wild-type, respectively; the MDA content were significantly decreased by 46.59%, 41.36% and 37.16% in OE-11, OE-7 and OE-12 compared with wild-type, respectively. The results showed that the content of Pro in transgenic lines increased significantly under stress (Fig. 11A), but the MDA content of transgenic lines was significantly lower than those of wild-type under stress (Fig. 11B). This conclusion indicates that the membrane system of cells in transgenic lines was protected, the degradation of protein in cells slowed down, and the adaptability to stress became stronger. We could infer that the overexpression of the *SpsNAC042* gene reduced the damage to plant cells under osmotic stress and made transgenic lines improved tolerance.

### Upregulated and downregulated expression of *MPK6*, *SOS1*, *HKT1*, *P5CS1* and *PRODH1* genes in transgenic *P. hopeiensis* under stress

**Fig. 12.**
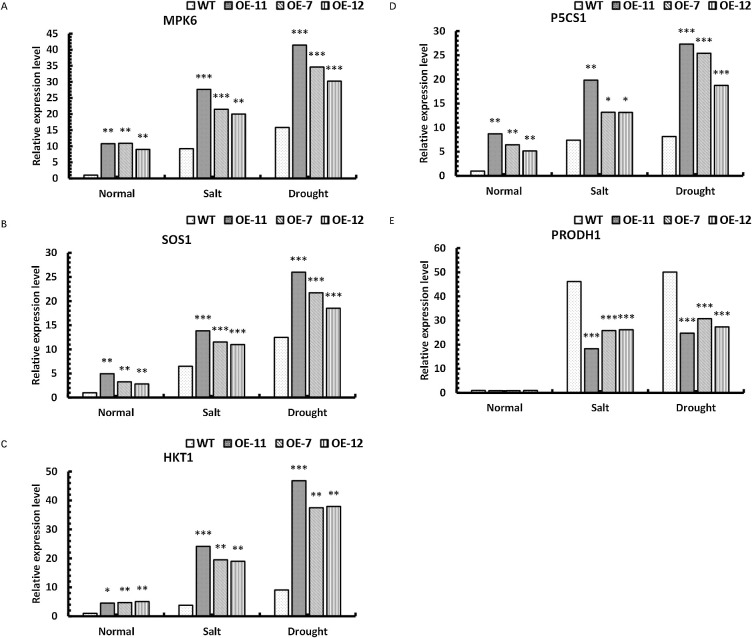
Expression analysis of *MPK6*, *SOS1*, *HKT1*, *P5CS1* and *PRODH1* genes in *P. hopeiensis* under salt and drought stress. (A) The relative expression level of *SOS1* in *P. hopeiensis*; (B) The relative expression level of *MPK6* in *P. hopeiensis*; (C) The relative expression level of *HKT1* in *P. hopeiensis*; (D) The relative expression level of *P5CS1* in *P. hopeiensis*; (E) The relative expression level of *PRODH1* in *P. hopeiensis*. Values are the mean ± standard deviation of three independent experiments. The significance of differences was determined using t-test and F-test. *** p < 0.001, extremely significant; ** 0.001 ≤ p < 0.01, very significant; * 0.01 ≤ p < 0.05, significant.

To analyze the possible regulatory pathways of *SpsNAC042* gene, changes in the expression levels of *MPK6*, *SOS1*, *HKT1*, *P5CS1* and *PRODH1* were examined. Under salt stress, the *MPK6* relative expression levels were significantly increased by 199.39%, 132.91% and 116.43% in OE-11, OE-7 and OE-12 compared with wild-type, respectively; the *SOS1* relative expression levels were significantly increased by 113.46%, 77.62% and 69.33% in OE-11, OE-7 and OE-12 compared with wild-type, respectively; the *HKT1* relative expression levels were significantly increased by 532.66%, 411.06% and 397.13% in OE-11, OE-7 and OE-12 compared with wild-type, respectively; the *P5CS1* relative expression levels were significantly increased by 168.46%, 78.03% and 77.74% in OE-11, OE-7 and OE-12 compared with wild-type, respectively; the *PRODH1* relative expression levels were significantly decreased by 60.32%, 44.00% and 43.25% in OE-11, OE-7 and OE-12 compared with wild-type, respectively. Under simulated drought stress, the *MPK6* relative expression levels were significantly increased by 161.97%, 118.73% and 90.94% in OE-11, OE-7 and OE-12 compared with wild-type, respectively; the *SOS1* relative expression levels were significantly increased by 108.20%, 74.10% and 48.30% in OE-11, OE-7 and OE-12 compared with wild-type, respectively; the *HKT1* relative expression levels were significantly increased by 416.98%, 313.99% and 318.73% in OE-11, OE-7 and OE-12 compared with wild-type, respectively; the *P5CS1* relative expression levels were significantly increased by 234.81%, 211.37% and 129.60% in OE-11, OE-7 and OE-12 compared with wild-type, respectively; the *PRODH1* relative expression levels were significantly decreased by 50.62%, 38.65% and 45.38% in OE-11, OE-7 and OE-12 compared with wild-type, respectively. These results indicate that the expression levels of *MPK6*, *SOS1*, *HKT1* and *P5CS1* in transgenic lines were significantly increased, but the expression level of *PRODH1* in transgenic lines was significantly decreased (Fig. 12). Therefore, *SpsNAC042* promoted the expression of the *MPK6*, *SOS1*, *HKT1* and *P5CS1*, but repression of *PRODH1* gene expression maintained the ion balance in plants and activated a series of metabolic reactions to alleviate the damage caused by stress to plants.

## Discussion

### *SpsNAC042* gene promotes the root development of plants

*SpsNAC042* gene promotes the growth and development of plant roots. In our study, the root number, root length and underground fresh weight of *SpsNAC042* transgenic lines increased significantly. Xie first proposed that *NAC1* has been proven was a lateral root regulatory factor, and its expression was related to auxin signaling pathway during lateral root development (Xie *et al.* 2000). It was found that GUS driven by the upstream regulatory region of *NAC1* was specifically expressed in root meristem, lateral root primordium base and lateral root, which may be involved in auxin and gibberellin signaling pathways to promote lateral root development (Wang *et al.* 2006a, 2006b). *NAC1* was proven to be independent of auxin in adventitious root regeneration of leaf explants, and it was speculated that *NAC* family genes may have different roles in organ formation and regeneration, while *NAC1* may have roles in lateral root and adventitious root formation in response to different upstream regulators (Chen *et al.* 2016). According to the above studies on *NAC1* gene function verification and regulation mechanism, it has been proved that *NAC1* transcription factor can positively regulate the lateral root development of plants. In our study, we also demonstrated that *SpsNAC042* gene promotes root development, the increase in root number, root length and underground fresh weight in transgenic lines was significantly higher than those of wild-type. It was further verified that *NAC042* gene also promoted lateral root development in woody plants.

### Effect of *SpsNAC042* gene on leaf morphological development of plants

We found the leaves of *SpsNAC042* transgenic lines showed curled phenotype. Then we think *SpsNAC042* gene influences morphological development of transgenic poplar leaves. In *Arabidopsis*, the overexpression of *TaNAC1* gene would cause the transgenic plants to have abnormal leaf development and slow growth, plant dwarfing and stem fusion (Ai *et al.* 2016). The overexpression of *ATAF2* gene in *Arabidopsis* led to leaf yellowing and dwarfing of transgenic plants. The reason may be that *ATAF2* gene activated the expression of *NIT* gene which changed the synthesis of auxin in transgenic plants, thus affecting the development of plants (Huh *et al.* 2012). The above studies confirmed that genes have a significant impact on the morphological development of different organs by regulating hormone levels in plants. We can also speculate that the leaf rolling of transgenic poplars is the result of plant hormone response. The poplar leaves overexpressing *SpsNAC042* showed an inward curling phenotype, which may also be due to the influence of plant gravity regulation, but there is no relevant research to prove it. In our study, although *NAC042* transgenic lines have different degrees of abnormal leaf development, due to too many factors affecting plant leaf morphogenesis, whether the *SpsNAC042* gene is directly involved in regulating plant leaf morphogenesis needs to be further confirmed.

### *SpsNAC042* enhances tolerance of plants to salt and simulated drought stress

Our study confirmed the *SpsNAC042* showed improved tolerance to salt and simulated drought stress. Yue found that the *StNAC1*-overexpressing transgenic tobacco with salt-treated had more proline content, and accumulated less ROS with higher seed germination rate and green leaf rate, which improved the salt tolerance of transgenic tobacco (Yue *et al.* 2021). *HcNAC1* has been identified to play an important regulatory role in response to drought stress in *Hibiscus cannabinus* (Deng *et al.* 2021). *PeNAC1* can be used to improve the salt tolerance and quality of oat through molecular breeding (Liang *et al.* 2021). The quantitative results showed that the expression of *AcoNAC1* gene in pineapple was induced by low temperature and drought stress, which was involved in the stress response of pineapple (Tan *et al.* 2021). In combination with the above studies, we found that the ability of the *NAC1* gene to resist abiotic stresses has gradually been verified in more plants. Our results showed that under salt and simulated drought treatment, the aboveground fresh weight, total chlorophyll content, SOD, POD, CAT activity and Pro content of *SpsNAC042*-overexpressing transgenic poplar were significantly higher than those of wild-type, and MDA content was significantly lower than that of wild-type. Under stress, excessive NaCl and PEG destroy the chlorophyll structure, thereby increasing the activity of related chlorophyll enzymes and ultimately hindering chlorophyll synthesis (Gao *et al.* 2018). However, in our study, the total chlorophyll content of the transgenic lines under stress was higher, the plant damage was lighter, and the tolerance was stronger.

We further determined three different antioxidant enzyme activities (SOD, POD and CAT) and MDA content in *SpsNAC042*-overexpressing transgenic poplar. The analysis of SOD, POD and CAT activities showed that the overexpressing lines produced more SOD, POD and CAT enzymes under stress than control group, which could eliminate reactive oxygen species and weaken the toxic effect of H_2_O_2_ on plant cells, so transgenic lines could protect the plasma membrane system, thereby enhanced the ability of plants to resist stress. However, the MDA content in transgenic plants was significantly lower than that of wild-type, which directly reflected *SpsNAC042*-overexpression poplar had less damage to biofilm structure and get back to normal physiological and biochemical processes soon. The above analysis results showed that the *SpsNAC042* played a positive regulatory role in salt and drought resistance, which made up for the research gap of *SpsNAC042* gene in woody plants and provided more choices for the salt-tolerant and drought-tolerant tree species.

### Regulatory mechanism of Na^+^ transporter pathway, proline synthesis and decomposition genes by *SpsNAC042* in alleviating stress

We analyzed the expression changes of a series of genes related to stress regulatory mechanisms such as *MPK6*, *SOS1*, *HKT1*, *P5CS1* and *PRODH1*. The *SpsNAC042* gene enhanced the expression of the *MPK6*, *SOS1*, *HKT1* and *P5CS1* genes, but inhibited *PRODH1* gene expression in transgenic poplars. MPK6 is a mitogen-activated protein kinase, and *MAPK* cascade pathway can be activated by various stresses and plays an important role in the response of plants to abiotic stresses (Zhang and Liu 2001). *SOS* pathway is a signal transduction pathway of abiotic stresses in plants that encodes plasma membrane Na^+^/H^+^ reverse transporter and is closely related to salt tolerance of plants (Zhu 2000). Its main function is responsible for intracellular Na^+^ efflux (Qiu *et al.* 2002). In the *SOS* pathway, MAPK6 (mitogen-activated protein kinase 6) is also involved in this signal transduction pathway. *MAPK6* binds to phosphatic acid and is then activated. Activated *MAPK6* binds to *SOS1* and completes the activity of Na^+^ efflux from cells (Yu *et al.* 2010). *HKT1* plays a role in plant physiological Na^+^ toxicity (Rubio *et al.* 1995). *P5CS* is a key enzyme in proline biosynthesis (Kishor *et al.* 2005). *PRODH* is a rate-limiting enzyme in the proline decomposition pathway (Funck *et al.* 2010). *MPK6* (Yu *et al.* 2010), *SOS1* (Chang 2019) and *HKT1* (Berthomieu *et al.* 2003) play a crucial role in plant tolerance to salt. *P5CS1* gene in *Arabidopsis* is induced by high salt and drought stress (Yoshiba *et al.* 1995). The antisense inhibition of *PRODH* gene improves the drought and salt tolerance of *Arabidopsis* (Nanjo *et al.* 1999). In our study, the expression of *MPK6*, *SOS1*, *HKT1* and *P5CS1* genes were significantly increased under salt stress and drought stress, and the expression of *PRODH1* gene was significantly decreased under stress, indicating that *SpsNAC042* gene may positively regulate *MPK6*, *SOS1*, *HKT1* and *P5CS*1 genes, and negatively regulate *PRODH1* gene, which contribute to ion balance within plants under stress, and accumulate proline content, maintain plant stability and enhance plant resistance.

### Relationship between root development and tolerance improvement in transgenic lines

The overexpression of *SpsNAC042* gene has a significant effect on the increase of plant root number and root length. The developed root system is an important guarantee for plants to obtain water and nutrients, and it is also a direct contact part of the soil. In the case of soil water deficit, the growth and development of plants depend on the water stored in the soil, which makes the roots grow rapidly under stress conditions to absorb water (Price *et al.* 2002). Therefore, the root number and length of plants are closely related to the establishment of plant tolerance. With the aggravation of drought stress, *Potentilla ansrina* and *Potentilla bifurca* responded to the damage caused by stress by increasing total root length, total surface area, volume and average root diameter (Zhang 2020). Under drought stress, root tip number, root dry weight, total root length, total root surface area and xylem area, as well as vascular bundle diameter and area of vascular bundles and bast in roots were significantly higher in strongly drought-tolerant alfalfa (Longzhong) than in medium drought-tolerant (Longdong) and drought-sensitive (Gannon No.3) alfalfa (Zhang *et al.* 2019). All of the above studies have shown that plants can improve water transport capacity and ensure plant survival under stress conditions by altering root morphology and internal conduction tissues. In our study, the increase in the root number and root length of *SpsNAC042*-overexpressing poplar may absorb more water for plants in environmental adversity to maintain plant vitality and make an important contribution to plant resistance to stress. Therefore, *SpsNAC042* gene may directly or indirectly improve plant resistance by regulating the root number and root length.

In addition, although *SpsNAC042*-overexpressing poplar had well-developed roots, transgenic poplar was somewhat stunted under non-stress condition. That maybe imply *SpsNAC042* influenced the height growth of overexpression lines. So we are considering it would be a good solution that root-specific expression vectors are used to drive the *SpsNAC042* gene in poplar transformation. Then well-developed root system will provide more nutrients and water for plant growth, and the aboveground part of transgenic poplar would escape from the developmental disadvantage. Alternatively, grafting may be a viable option to eliminate *SpsNAC042* negative effect on the aboveground part of transgenic poplar. *SpsNAC042*-overexpressing poplar could be used as the rootstock in grafting of near-source species. With the rapid development of plant genetic engineering technology, resistant plants bred using transgenic technology will be one of the important choices for future afforestation projects. In conclusion, the study of *SpsNAC042* provides a research basis for the subsequent excavation of *NAC* genes and provides ideas for exploring other resistance genes from *S. psammophila*.

### Conclusion

The *NAC042* gene was cloned from *S. psammophila* and eventually obtained in *SpsNAC042*-overexpressing *P. hopeiensis*. Expression of the *SpsNAC042* gene promoted root development, affected leaf morphology, and enhanced tolerance to salt and simulated drought stresses. Under non-stress, the root length of *SpsNAC042*-overexpressing *P. hopeiensis* increased significantly and the leaves showed curled phenotype. Under salt and drought stress, the overexpression of *SpsNAC042* gene effectively increased SOD activity, POD activity, CAT content and Pro content, and reduced MDA accumulation to maintain the normal physiological state of plants to resist the damage caused by stress. At the same time, it was found that *SpsNAC042* gene could positively regulate the expression of *SOS1*, *MPK6*, *HKT1* and *P5CS1* genes, and negatively regulate the expression of *PRODH1* gene. *SpsNAC042* plays an important role in the regulation of Na^+^ transporter pathway, proline synthesis and decomposition during stress.

## Author Contribution Statement

FLJ was a major contributor in writing the manuscript. WDS analyzed the data and make figures. YXW completed the vector construction. WJM assisted in the sample collection. YFQ, SGR, Alatengsuhe, ZL provided plant material (*S. psammophila*). ZGS and YHF provided guidance and advice on stress methods in earlier stages of this study.

## Supplementary Material

Supplemental Figures

## Figures and Tables

**Table 1.** The related genes primer of qPCR

Primer name	Primer sequence (5ʹ-3ʹ)
*SpsNAC042-F*	ATGAGCAACATAAGTTTTGTGGAGGC
*SpsNAC042-R*	ATTGTGGCAGAAGAGAAACCCT
*qN1-F*	GCAACTGGACTAAGAACGAATCG
*qN1-R*	ACGACCCAGTACTGGCCCTT
*UBQ-F*	GCATCATCACAATCACTCTCCGA
*UBQ-R*	ACCACCAGCCTTCTGGTAAA
*ACT-F*	AAACTGTAATGGTCCTCCCCG
*ACT-R*	GCATCATCACAATCACTCTCCGA
*SOS1-F1*	GGCTGTTGTTGCTCTGTTGA
*SOS1-R1*	TTATGGCACCCGAGGTAAAG
*MPK6-F1*	CTGCAAACGTCCTGCATAGA
*MPK6-R1*	AACACACAACCCACTGACCA
*HKT1-F1*	TGGTTCAGTGCCTGTTGTTC
*HKT1-R1*	CATCCTTGCACGAGCTATCA
*P5CS1-F1*	GTGTTGGCACCCTCTTTCAT
*P5CS1-R1*	CATCAGCTACGTCCAGCAAA
*PRODH1-F1*	ATGGCACGATTCAAGCCTAC
*PRODH1-R1*	TTCAAGCATGAACGAAGCAC
