# Supplementary material for: Effects of SpsNAC042 transgenic Populus hopeiensis on root development, leaf morphology and stress resistance
Source: Breed Sci. 2023 Apr 13;73(2):180–92. doi: 10.1270/jsbbs.22079 (PMC10316303; doi:10.1270/jsbbs.22079)
Supplement: Supplementary file 1 — Supplemental Figures [file 73_180_s1.pdf]

Fan et al./Breeding Science
doi: 10.1270/jsbbs.22079

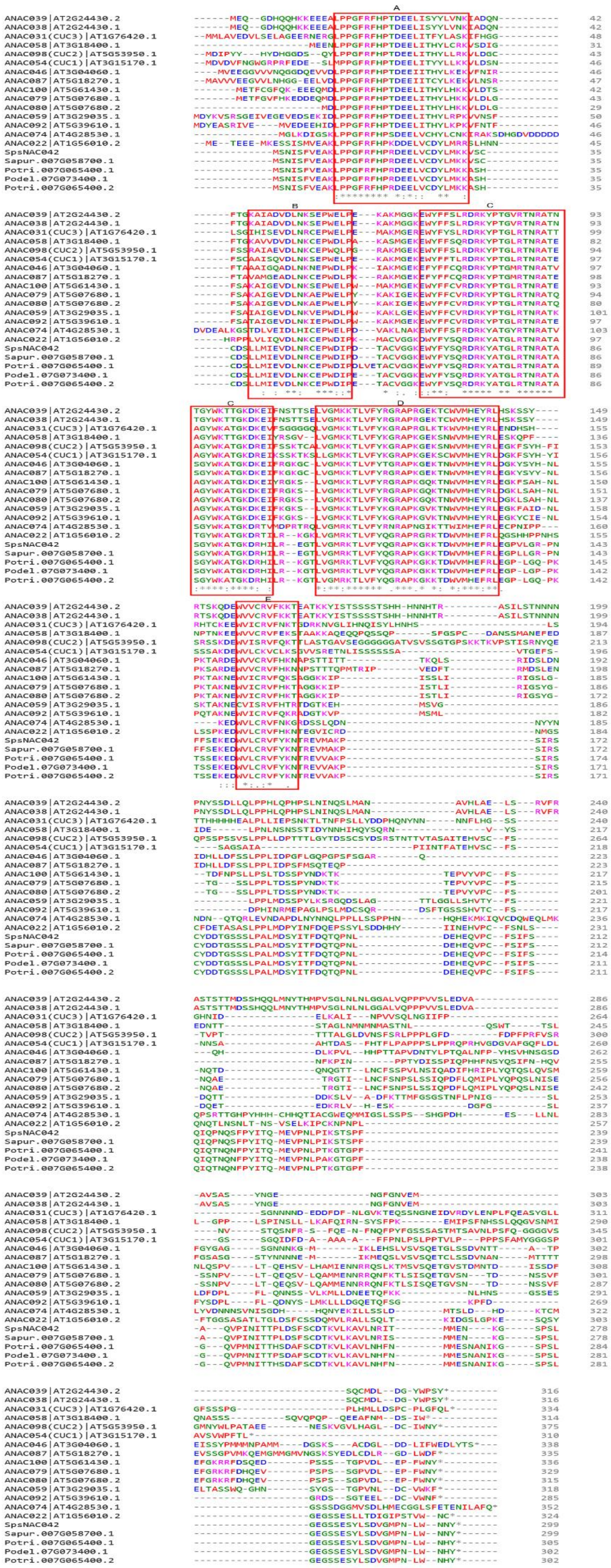

Supplemental Fig. 1. *NAC* transcription factor structure analysis of *Salix*, *Arabidopsis*, and *Populus*.

```
1       ATGAGCAACATAAGTTTTGTGGAGGCAAAATTGCCACCAGGGTTCAGGTTCCATCCAAGA
1        M  S  N  I  S  F  V  E  A  K  L  P  P  G  F  R  F  H  P  R
61      GATGAAGAGCTTGTGTGTGACTACTTGATGAAGAAGGTTTCCTGCTGTGACTCCCTTCTT
21       D  E  E  L  V  C  D  Y  L  M  K  K  V  S  C  C  D  S  L  L
121     ATGATAGAAGTTGACCTCAACAGGTGTGAGCCGTGGGATATTCCTGATACGGCATGCGTG
41       M  I  E  V  D  L  N  R  C  E  P  W  D  I  P  D  T  A  C  V
181     GGAGGCAAGGAATGGTACTTTTATAGCCAAAGAGATCGTAAATACGCAACTGGACTAAGA
61       G  G  K  E  W  Y  F  Y  S  Q  R  D  R  K  Y  A  T  G  L  R
241     ACGAATCGAGCAACAGCATCTGGATATTGGAAGGCCACAGGGAAGGACAGACATATCCTA
81       T  N  R  A  T  A  S  G  Y  W  K  A  T  G  K  D  R  H  I  L
301     CGTGAGGGAACCCTTGTTGGCATGAGAAAGACCTTGGTGTTCTACCAAGGTAGGGCACCT
101      R  E  G  T  L  V  G  M  R  K  T  L  V  F  Y  Q  G  R  A  P
361     AAAGGGAAAAAAACTGATTGGGTCATGCATGAGTTTCGGCTTGAAGGGCCAGTACTGGGT
121      K  G  K  K  T  D  W  V  M  H  E  F  R  L  E  G  P  V  L  G
421     CGTCCCAATTTTTTTTCAGAGAAGGAAGACTGGGTTTTATGTCGAGTGTTCTATAAAAAC
141      R  P  N  F  F  S  E  K  E  D  W  V  L  C  R  V  F  Y  K  N
481     ACTAGAGAAGTTATGGCCAAACCTAGCATAAGAAGCTGCTATGATGACACTGGCTCTTCA
161      T  R  E  V  M  A  K  P  S  I  R  S  C  Y  D  D  T  G  S  S
541     TCTTTGCCTGCGTTAATGGATTCATATATCACTTTTGACCAAACTCAACCCAATTTAGAT
181      S  L  P  A  L  M  D  S  Y  I  T  F  D  Q  T  Q  P  N  L  D
601     GAGCACGAGCAAGTGCCCTGCTTCTCCATTTTCTCTCAAATCCAACCCAACCAAAGTTTC
201      E  H  E  Q  V  P  C  F  S  I  F  S  Q  I  Q  P  N  Q  S  F
661     CCATACATCACTCAAATGGAAGTGCCAAATTTACCTATAAAGAGTACAAGCCCATTTGCT
221      P  Y  I  T  Q  M  E  V  P  N  L  P  I  K  S  T  S  P  F  A
721     CAAGTACCTATAAATATCACCACACCTCTAGACTCTTTTTCTTGTGATACAAAGGTACTA
241      Q  V  P  I  N  I  T  T  P  L  D  S  F  S  C  D  T  K  V  L
781     AAAGCTGTTTTGAATCGCATTACCATGATGGAAAACAAAGGGTCACCAAGCTTAGGAGAA
261      K  A  V  L  N  R  I  T  M  M  E  N  K  G  S  P  S  L  G  E
841     GGTAGTTCAGAAAGCTACTTATCTGATGTGGGCATGCCCAACTTATGGAATAATTATTGA
281      G  S  S  E  S  Y  L  S  D  V  G  M  P  N  L  W  N  N  Y  *
```

Supplemental Fig. 2. *SpsNAC042* gene sequence and its translated amino acid sequence.
